# Effect of combination treatment of S–amlodipine with peroxisome proliferator-activated receptor agonists on metabolic and cardiovascular parameters in Zucker *fa/fa* rats

**DOI:** 10.1186/1758-5996-6-45

**Published:** 2014-03-28

**Authors:** Bhagat Singh, Ganesh V Sangle, Jeya Murugan, Rinku Umrani, Subhasis Roy, Onkar Kulkarni, Arvind Semwal, MK Unnikrishnan, Mukul Jain

**Affiliations:** 1 Department of Clinical Neuroscience, University of Calgary, 3330 Hospital Drive, NW, Calgary, Alberta T2N4N1, Canada; 2 Department of Physiology, University of Manitoba, Winnipeg, Canada; 3 Department of Pharmacology, Zydus Research Centre, Ahmedabad, India; 4 Department of Pharmacology, Manipal College of Pharmaceutical Sciences, Manipal University, Karnataka, India; 5 Department of Pharmacy, BITS Pilani Hyderabad campus, Jawahar Nagar, Hyderabad, India

**Keywords:** Calcium channel blockers, Peroxisome proliferator-activated receptors (PPARs), Type 2 diabetes, Dyslipidemia, Hypertension

## Abstract

**Background:**

Type 2 diabetes is a complex metabolic disorder characterized by hyperglycemia, impaired glucose tolerance and insulin resistance associated with dyslipidemia and hypertension. The available drugs are not sufficiently efficacious in reducing cardiovascular risk and restoring normal glucose metabolism associated with type 2 diabetes as a mono- or a combination therapy. The present study examined the combined effects of an antihypertensive (S-Amlodipine) and an insulin-sensitizing agent, peroxisome proliferator-activated receptor (PPAR) agonists (Pioglitazone and Ragaglitazar), on cardiovascular risk factors in aged diabetic and insulin-resistant Zucker *fa/fa* rats.

**Methods:**

Following combination treatment for 14 days, blood pressure (BP), serum glucose, total cholesterol and triglycerides were measured. Aortic ring study was conducted to determine the effect of combination treatments on phenylephrine-induced vasoconstriction and acetylcholine (Ach)-induced vasorelaxation.

**Results:**

In combination, S-Amlodipine and Pioglitazone significantly reduced blood glucose (115.1 ± 6.6 vs. 81.7 ± 4.2), BP (184.4 ± 5.0 vs. 155.1 ± 5.0), serum triglycerides (362.5 ± 47.5 vs. 211.1 ± 23.7) and glucose intolerance when compared with vehicle treated Zucker *fa/fa* rats. Similar results were observed with the combination of S-Amlodipine and Ragaglitazar (Triglycerides, 362.5 ± 47.5 vs. 252.34 ± 27.86; BP, 184.4 ± 5.0 vs. 159.0 ± 8.0) except for serum glucose. ACh-induced vasorelaxation in aortic rings was also superior with both of the combinations compared to individual treatment. Furthermore, there was less body weight gain and food intake with S-Amlodipine and Pioglitazone combination in Zucker *fa/fa* rats. S-Amlodipine itself caused significant reduction in glucose (115.1 ± 6.6 vs. 89.7 ± 2.7) and BP (184.4 ± 5.0 vs. 156.1 ± 4.0) with improvement in insulin sensitivity observed through oral glucose tolerance test.

**Conclusions:**

The results suggest that a combination of PPAR agonists and S-Amlodipine has partial benefits in improving the cardiovascular risk factors such as reduction in triglyceride levels, associated with chronic type 2 diabetes, and therefore may be utilized as an approach for addressing some of these devastating metabolic syndrome complications.

## Introduction

Diabetes mellitus [1] is characterized by hyperglycemia resulting from defects in insulin secretion, insulin action or both. Type 2 DM is the most common form of diabetes [2]. According to the World Health Organization, up to 3 billion people will be affected with DM by the year 2025. Type 2 DM is a heterogeneous, progressive disorder initially characterized by impaired glucose tolerance and compensatory hyperinsulinemia, which in the later stages, develops severe insulin resistance and impaired beta cell function [2,3]. This is associated with hyperglycemia, dyslipidemia, hypertension, obesity and in the long term, micro-and macro-vascular complications leading to increased mortality [4]. Hypertension (blood pressure [BP] ≥140/90 mmHg) is an extremely common condition in diabetes, affecting around 20 to 60% of diabetic patients [5]. Unfortunately, none of the available drugs for clinical use have proved sufficiently efficacious in reducing cardiovascular risk and restoring normal glucose metabolism in mono or in combination therapy as the disease progresses [6].

Peroxisome proliferator-activated receptors (PPARs) are transcription factors belonging to the superfamily of nuclear receptors. Three isoforms (alpha, beta and gamma) have been identified [7]. PPAR-gamma agonists, also known as thiazolidinediones (TZDs), such as Pioglitazone and Rosiglitazone increase insulin sensitivity, reduce levels of blood glucose, insulin and triglycerides with a concomitant reduction in BP and improvement in endothelial function [8,9]. PPAR-alpha agonists in the form of fibrates have been in use for the treatment of dyslipidemia [10]. PPAR-alpha and PPAR-gamma were empirically discovered owing to their ability to improve insulin sensitivity and lipidemia, respectively in rodents [11,12]. Endothelium dependent relaxation is impaired in diabetic conditions [13]. Zucker *fa/fa* rats are insulin resistant, hyperglycemic and have blunted endothelium vasorelaxation [14]. In addition, a considerable number of *in-vitro* and *in-vivo* studies reported impact of TZDs on the cardiovascular system including reduction in BP, restoration of blunted endothelium-mediated vasodilation, attenuation of sympathetic overactivity, inhibition of intracellular Ca^2+^ increase, and proliferation of vascular smooth muscle cells [13,15-17]. Consequently, agonists with dual PPAR-alpha and gamma activity would potentially have beneficial effects superior to those obtained with drugs activating either of the PPAR subtypes alone. Ragaglitazar, a non-TZD compound has a combined PPAR-alpha/gamma agonist activity both *in-vitro* and *in-vivo* and reported efficacious in lowering blood glucose and lipid profile [4].

Calcium channel blockers (CCBs) are one of the most widely used drugs for the treatment of hypertension associated with type 2 DM [18]. Some preliminary previous studies have demonstrated reduced levels of blood glucose and lipid parameters in different rat models as well as in humans with CCBs, but results were not significant [19,20]. The impact of combination treatment of CCB and PPAR agonist in Zucker *fa/fa* rats remains unknown.

The present study was conducted to evaluate the impact of combination treatments of CCB and PPAR agonist (S-Amlodipine + Pioglitazone and S-Amlodipine + Ragaglitazar) on biochemical and cardiovascular parameters in aged Zucker *fa/fa* rats.

## Materials and methods

### Materials

Ragaglitazar and Pioglitazone were synthesized and obtained from the chemistry department, Zydus Research Center, Ahmedabad, India. Phenylephrine (PE), L-NAME and Ach were obtained from Sigma (USA). All other reagents were of analytical grade. PE and ACh were made up as fresh base solutions in physiological salt solution.

### Animals and experimental design

Eighteen week old male Zucker *fa/fa* rats were obtained from Charles River Laboratories (USA) and housed at an ambient temperature of 22 ± 2°C. Animals were maintained on a 12-h day/night cycle with *ad libitum* access to standard lab diet (National Institute of Nutrition, Hyderabad, India). The Institutional Animals Ethical Committee Guidelines approved the methods and procedures described in the present report. Animals were divided into 6 different groups (n = 7), control vehicle (0.25% Tween 80), S-Amlodipine (1 mg/kg), Pioglitazone (6 mg/kg), Ragaglitazar (1 mg/kg), S-Amlodipine (1 mg/kg) + Pioglitazone (6 mg/kg), S-Amlodipine (1 mg/kg) + Ragaglitazar (1 mg/kg) by oral gavage once-daily for 14 days.

### Blood pressure measurement

BP was measured in conscious state using tail-cuff method (NIBP-4 Columbus instruments, Ohio, USA) after pre-warming the tail for 15 min at 30°C. To ensure the reproducibility, 10 measurements were made and the mean of the measurements calculated at day 0 and day 14. The animals were acclimatized for 15 min. before the experiment on the experimental day. During BP measurement, the animals were calm and tail was motionless. The time constant was set to 120 seconds.

### Body weight and food intake

Body weight and feed consumption were recoded weekly to monitor any changes. The amount of feed consumption (in g) over the one week period was recorded for each treatment group.

### Blood collection and analysis of serum glucose, lipid profile, creatinine, and electrolytes

Animals were kept for overnight fasting and blood was collected by retro-orbital puncture using local anesthesia. Serum was separated immediately and levels of glucose, triglyceride (TG), total cholesterol (TC) and creatinine were analyzed by a Liasys, biochemical analyzer (AMS, Italy).

### Oral Glucose Tolerance Test (OGTT)

A glucose load of 5 g/kg/3 ml was given orally to overnight fasted (16–18 h) animals and blood was collected from retrorbital sinus at the subsequent interval of 0, 30, 60 and 120 min. The serum was separated immediately and the samples were analyzed for glucose using a commercial kit (Pointe scientific, USA) based on the GOD-POD principle using Spectramax 190 Microplate Spectrophotometer.

### Aortic ring study for the assessment of vascular reactivity

Thoracic aortas were isolated and placed in cold physiological salt solution and cut into 4 mm cylindrical segment after cleaning the connective tissue. Blood vessels were mounted in a 20-ml jacketed organ bath (Expermetria Ltd., Hungary) containing physiological salt solution. The lumen of each vessel was cannulated with wire hooks; one end of hook was fastened to stationary glass rod and the other to an isometric force transducer (Experimetria Ltd., Hungary) interfaced to computerized data acquisition program (Acqnowledge v 3.5.7, Biopac Systems, USA).

Physiological salt solution was maintained at 37°C and aerated with 95% O_2_ – 5% CO_2_ throughout the experiment. Aortic rings were placed under resting tension (1.5 g) and equilibrated for ~60 min with washing. A cumulative dose response of PE (10 nmol/l – 10 μmol/l) in endothelium intact tissues was recorded followed by a cumulative dose response to ACh (30 nmol/l-30 μmol/l) in PE precontracted aorta. Isometric tension was measured as grams of force and then normalized to maximal contraction to PE. Vasoconstriction in response to PE was expressed as a percentage of the maximal tension generated in response to 10 μM PE. Relaxation in response to each concentration of ACh was expressed as the percentage reduction from the maximal tension generated in response to PE (1 μM) alone.

### Statistical analysis

All data are presented as means ± standard error mean [SEM]. Unless stated, statistical analyses were done by one-way ANOVA followed by Dunnet’s significant difference test for multiple comparisons using Graph Pad Prism software 4.0. Differences were considered statistically significant when p < 0.05.

## Results

### Effect on body weight and food intake

**Figure 1:**
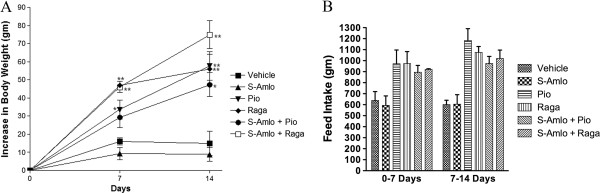
**Effect of treatments on body weight and feed intake.** Effect of combination of S-Amlodipine with Ragaglitazar and Pioglitazone on body weight **(A)** and food intake **(B)** after 14 days of treatment in *fa/fa* rats. Data are shown as means ± S.E.M. n = 7 per group. *Significantly different from the control (*p < 0.05, **p < 0.01).

Individual treatment with Pioglitazone and Ragaglitazar showed significant increase in body weight compared to vehicle group. S-Amlodipine alone or in combination with Pioglitazone showed less gain in body weight compared to that of individual treatment. The decrease in body weight was partially correlated with reduction in food intake (Figure 1a and b).

### Effect on serum glucose, lipid profile and creatinine

Serum glucose was reduced significantly with S-Amlodipine, Pioglitazone and their combination (Table 1). Pioglitazone, Ragaglitazar and their combinations with S-Amlodipine showed significant reduction in serum TG levels. S-Amlodipine alone showed a non-significant trend towards reduction in TG levels. There was no change in TC levels with any of the treatment (Table 1). Serum creatinine levels were not significantly different between the treatments (Table 1).

**Table 1:** **Effect of combination of S-Amlodipine with Ragaglitazar and Pioglitazone on biochemical parameters in Zucker *fa/fa *** **rats**

	**Glucose (mg/dl)**	**TC (mg/dl)**	**TG (mg/dl)**	**Creatinine**
Control	115.18 ± 6.64	139.08 ± 14.55	362.56 ± 47.53	0.88 ± 0.08
S-Amlodipine	89.71 ± 2.72*	134.94 ± 12.89	267.75 ± 17.15	0.78 ± 0.12
Pioglitazone	90.60 ± 2.11*	146.50 ± 4.10	230.64 ± 18.81**	0.76 ± 0.04
Ragaglitazar	106.45 ± 1.55	133.58 ± 2.59	173.21 ± 15.70**	0.77 ± 0.08
S-Amlo + Pio	81.76 ± 4.24*^$^	131.97 ± 10.03	211.14 ± 23.78**	0.80 ± 0.05
S-Amlo + Raga	103.01 ± 7.48	148.34 ± 14.47	252.34 ± 27.86*	0.79 ± 0.04

Data are shown as means ± S.E.M. n = 7 per group. *significantly different from the control (*p < 0.05, **p < 0.01); (^$^p < 0.05; one-tailed ‘t’ test; Pio vs. Amlo + Pio). Amlo, Amlodipine; Pio, Pioglitazone; Raga, Ragaglitazar.

### Effect on oral glucose tolerance

**Figure 2:**
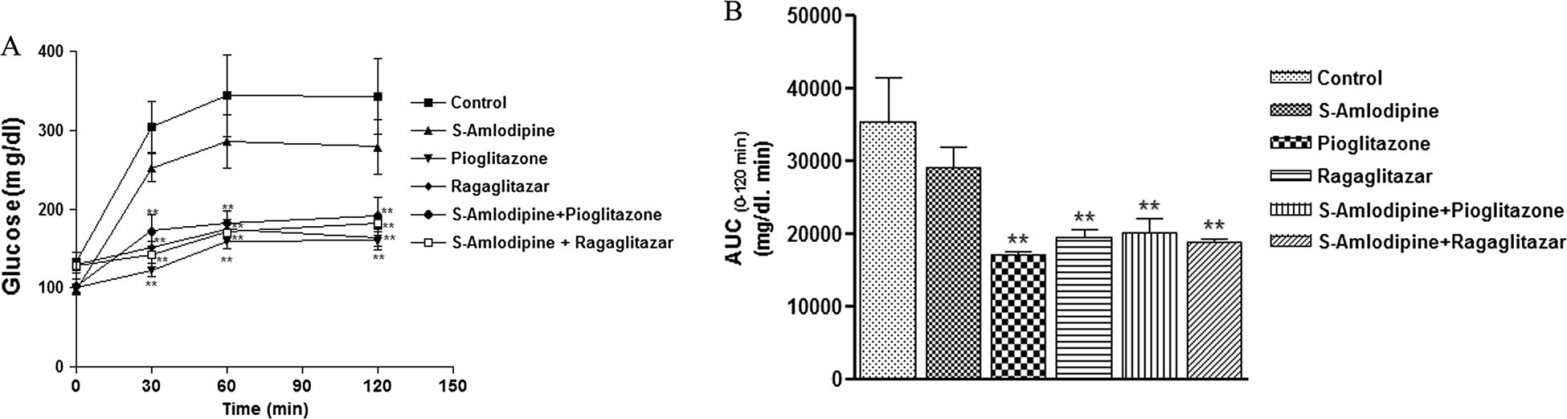
**Impact of treatments in oral glucose tolerance test (OGTT).** Impact of combination of S-Amlodipine with Ragaglitazar and Pioglitazone on glucose tolerance after 14 days of treatment in *fa/fa* rat. **A**. Glucose levels at different time points. **B**. Area under the curve (0–120 min) of OGTT. Data are shown as means ± S.E.M. n = 7 per group. *Significantly different from the control (*p < 0.05, **p < 0.01).

All the treatments including both combinations showed improvement in glucose tolerance. We found significant reduction in area under curve [21] with all the treatments except S-Amlodipine when compared with vehicle group, but this reduction was not synergistic in action on glucose tolerance test (Figure 2a and b). All the treatments including S-Amlodipine alone inhibited the rise in first phase insulin at 30 min post glucose load. At 2 h post glucose load, vehicle treated animals still had higher glucose levels whereas Pioglitazone, Ragaglitazar and their respective combination with S-Amlodipine brought the glucose levels closer to baseline.

### Effect on systolic blood pressure (SBP)

**Figure 3:**
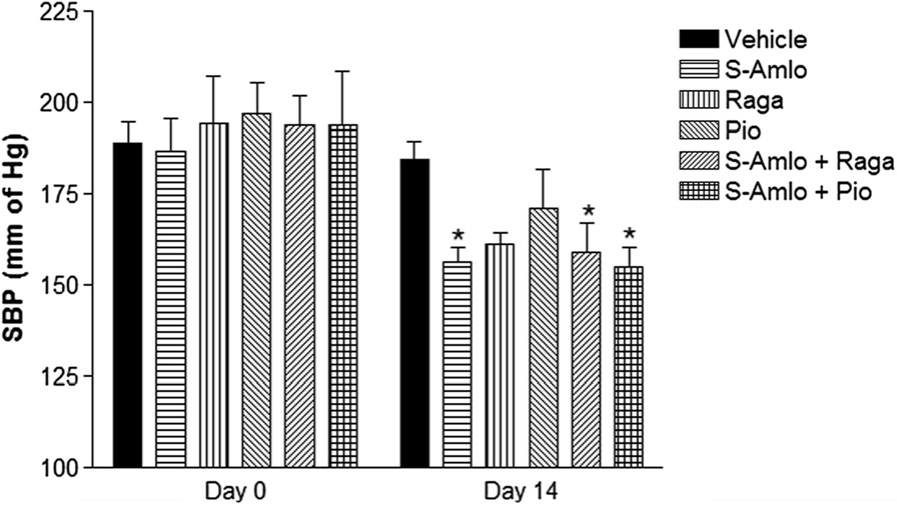
**Effect of treatments on systolic blood pressure (SBP).** Effect of combination of S-Amlodipine with Ragaglitazar and Pioglitazone on SBP after 14 days of treatment in *fa/fa* rats. Data are shown as means ± S.E.M. n = 7 per group. *Significantly different from the control (*p < 0.05).

We found significant reduction in SBP with S-Amlodipine alone or in combinations of S-Amlodipine with both Ragaglitazar and Pioglitazone. The overall reduction in SBP was not synergistic with combinations. It could be possible that the small reduction with Pioglitazone and Ragaglitazar was due to overall improvement in lipid and diabetic parameters. S-Amlodipine itself reduced BP up to a level where Pioglitazone and Ragaglitazar cannot advantage further at least for BP reduction (Figure 3).

### Effect on smooth muscle relaxation

**Figure 4:**
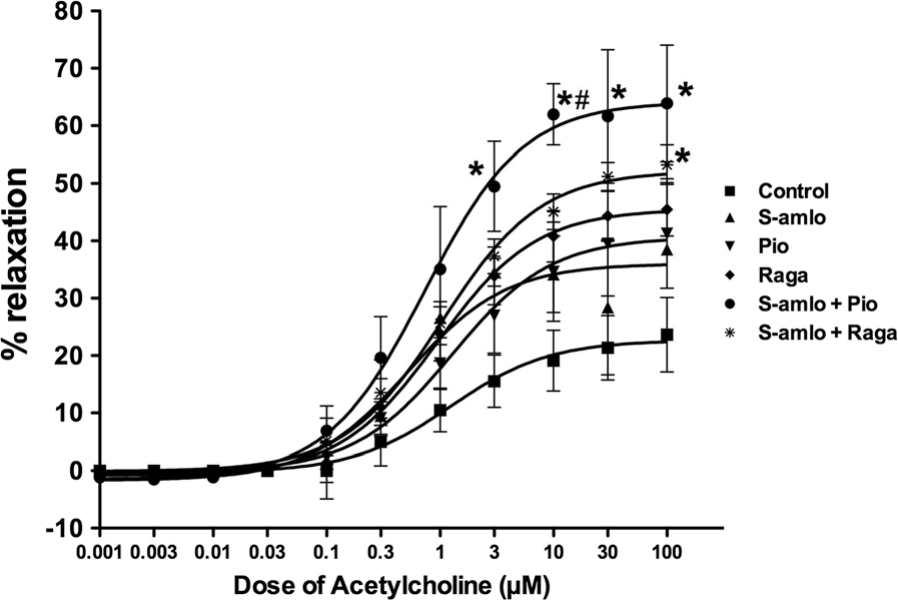
**Effect of treatments on ACh induced aorta relaxation.** Effect of combination of S-Amlodipine with Ragaglitazar and Pioglitazone on ACh induced relaxation on aorta of 14 days treated *fa/fa* rats. Data are shown as means ± S.E.M. *Significantly different from the control (*p < 0.05; #, p < 0.05, Pioglitazone and Amlodipine + Pioglitazone).

PE induced contraction was not altered with any of the treatments (data not shown). ACh induced relaxation was increased with all the treatment groups but it was more prominent with combination therapy. S-Amlodipine, Pioglitazone and Ragaglitazar have showed 30-40% vasorelaxation but it was 50–60% with the combination treatments (Figure 4). There was significant improvement in the endothelial relaxation with both of the combinations, although it was higher with the S-Amlodipine and Pioglitazone combination (Figure 4). The reduction in BP with combination therapy could be attributed to the increased aortic relaxation, as both BP reduction and aortic relaxation were greater with the combination therapy.

## Discussion

The major findings from this work are (1) S-Amlodipine cause reduction in BP, blood glucose and triglyceride levels, improves insulin sensitivity and induces less gain in body weight; (2) S-Amlodipine combined with Pioglitazone leads to significant decrease in serum glucose levels, induces less gain in body weight with simultaneous improvement in smooth muscle relaxation; (3) S-Amlodipine combined with Ragaglitazar shows further improvement in TG reduction; and (4) combination treatments has no synergistic impact on BP reduction and insulin sensitivity in Zucker *fa/fa* rats.

Metabolic syndrome and type 2 DM, characterized by obesity, insulin resistance, and dyslipidemia, have reached an epidemic proportion in developed societies. Blood glucose levels can be controlled pharmacologically to prevent the majority of complications associated with diabetes; however the current treatment regime does not adequately normalize the blood glucose level in type 2 DM patients [2]. TZDs improve insulin sensitivity; decrease circulating insulin levels and lower fasting blood glucose in diabetic individuals. These changes are associated with reversal of many of the components of the insulin resistance syndrome, including lowered triglycerides, increased high-density lipoprotein (HDL), decreased small dense low-density lipoprotein (LDL), reduced circulating plasminogen activator inhibitor-1 levels, and decreased BP. These observations suggest that reversal of the insulin resistance syndrome is associated with an improvement in the cardiovascular risk factors associated with insulin resistance [22]. Both hypertension and type 2 DM are multifaceted dynamic expressions of pathophysiological disequilibrium that are closely related by a number of common factors. The present study was conducted to evaluate combination therapy of PPAR agonists and CCB, S-Amlodipine as a possible treatment for metabolic syndrome and type 2 DM.

S-Amlodipine alone or in combination with Pioglitazone and Ragaglitazar showed significant reduction in SBP in Zucker *fa/fa* rats. Combinations did not show any synergistic effect on BP reduction, as this could be the maximum or saturation effect of S-Amlodipine alone. S-Amlodipine lowers both systolic and diastolic pressure by direct vasodilatation through blocking calcium channels and increases the heart rate by reflex tachycardia [19]. Individual treatments with Ragaglitazar and Pioglitazone showed a trend towards reducing BP, but it was not statistically significant. PPAR-gamma agonists activate renal sodium and water reabsorptive pathways, and lowers BP in normal rats [23]. Recent studies demonstrated reduction in BP with Pioglitazone or Ragaglitazar treatment, which was mediated through vasodilatation by increasing the nitric oxide (NO) release [13,16]. The present study demonstrated increased ACh induced relaxation in aortic rings of Zucker *fa/fa* rats treated for 14 days with combination treatment, which shows a better improvement in endothelial function with the combination therapy compared to individual treatments. The improved endothelial function could be one of the mechanisms for changes in BP. Recent studies have also identified PI3K-Akt pathway and hypoxia inducible factor -1 alpha and genetic basis other than nitric oxide as possible mechanisms for PPAR induced smooth muscle relaxation and improvement in endothelial dysfunction [24,25].

Weight gain associated with increased food intake is a major side effect of Pioglitazone treatment in both animal models and humans. PPAR-gamma increases feeding by reducing leptin levels, a hormone present in the hypothalamus that regulates satiety center [6]. Increase in body mass was partially due to increased feeding and perhaps reabsorption of sodium ions in collecting ducts, which could cause peripheral edema [1]. PPAR agonists (Pioglitazone and Ragaglitazar) showed significant weight gain, associated with increased food intake as previously reported [6]. Interestingly, S-Amlodipine showed lesser gain in body weight with reduced feed intake compared to vehicle group as supported by others in diabetic rats [26]. Surprisingly, S-Amlodipine combined with Pioglitazone showed lesser gain in body weight compared to the animals receiving Pioglitazone alone. This reduction in body weight was well correlated with reduction in food intake. The results suggest that combination treatment may have advantage in controlling weight gain in these animals.

Serum glucose levels were reduced significantly with S-Amlodipine and Pioglitazone individually and in combination. The decrease in blood glucose levels by S-Amlodipine could be due to the increase in insulin sensitivity as seen in OGTT, which in turn might be related to the decrease in cytosolic Ca^2+^ concentration [19,27]. Moreover, S-Amlodipine showed significantly smaller increase in peak serum glucose levels in OGTT after oral glucose load, which could be due to increased 1^st^ phase insulin secretion by S-Amlodipine or other possible mechanisms such as alteration in the beta-adrenergic activity that in turn influences the insulin release [28]. Both Pioglitazone and Ragaglitazar caused a reduction in serum glucose levels in various animal species by acting as insulin sensitizer [29]. The results of the present study demonstrated significant reduction in glucose levels with S-Amlodipine and Pioglitazone in combination. This is correlated with the increase in insulin sensitivity as the total area under the curve [21] in OGTT was reduced significantly and further reduction in glucose levels reaching to normal after 2 hour of glucose load with the combination treatment.

PPAR-alpha agonists are effective in reducing triglyceride (TG) and LDL levels with elevated HDL modulating various genes like lipoprotein lipase, apolipoprotein C-III, human apolipoprotein A-I and A-II [30]. A previous study demonstrated reduced levels of TG and free fatty acid (FFA) in normal and *fa/fa* rats induced by Pioglitazone [31]. The results of the present study suggest significant reduction in TG levels with Ragaglitazar treatment due to PPAR-alpha fraction, which was supported by previous reports [8]. S-Amlodipine showed a partial non-significant reduction in TG levels as supported by previous studies [19,26]. The authors suggested that the decrease in TG is due to the inhibition of intestinal chylomicron secretion and the enhancement of hepatic uptake of very low-density lipoprotein (VLDL) [21]. Both the combinations have demonstrated reduction in TG levels up to the same extent as individual treatments.

Jian et al. found that plasma creatinine levels were not significantly different, though creatinine clearance was significantly reduced by Rosiglitazone treatment, possibly indicating some fall in glomerular filtration rate. However, the urinary K^+^ to Na^+^ ratio was increased by Rosiglitazone treatment, indicating selective Na^+^ reabsorption in the kidney relative to K^+^[23]. The present study demonstrated increased Na^+^ excretion, and improved renal function as indicated by reduction in serum creatinine levels with individual treatments. This renal improvement accompanied with increased Na^+^ excretion may be one of the factors responsible for reduction in BP in diabetic rats [19,32]. It is well known that the Zucker *fa/fa* rats have abnormal renal function. It was reported that Pioglitazone and Troglitazone prevent glomerular dysfunction in diabetic rats by inhibition of the DAG-PKC-ERK pathway with improved renal function [33]. This improvement in renal function could be very useful in diabetes-associated complications.

## Conclusions

In conclusion, our results suggest that a calcium channel blockers such as S-Amlodipine combined with PPAR agonists, Pioglitazone or Ragaglitazar, could improve certain metabolic and cardiovascular risk factors in patients suffering from metabolic syndrome but further studies are warranted to validate the conclusions.

## Competing interests

The authors report no declarations of interest. The authors alone are responsible for the content and writing of the paper.

## Authors’ contributions

BS carried out most of the experimental work with designing, execution and analysis of the data. GS, JY and RU helped with the *in vitro* experiments. SR, OK and AS helped with the animal work. BS, MKU and MJ conceived, designed and wrote the manuscript. All authors read and approved the final manuscript.
